# Plasma membrane protein OsMCA1 is involved in regulation of hypo-osmotic shock-induced Ca^2+ ^influx and modulates generation of reactive oxygen species in cultured rice cells

**DOI:** 10.1186/1471-2229-12-11

**Published:** 2012-01-23

**Authors:** Takamitsu Kurusu, Daisuke Nishikawa, Yukari Yamazaki, Mariko Gotoh, Masataka Nakano, Haruyasu Hamada, Takuya Yamanaka, Kazuko Iida, Yuko Nakagawa, Hikaru Saji, Kazuo Shinozaki, Hidetoshi Iida, Kazuyuki Kuchitsu

**Affiliations:** 1 Department of Applied Biological Science, Tokyo University of Science, 2641 Yamazaki, Noda, Chiba 278-8510, Japan; 2 Research Institute for Science and Technology (RIST), Tokyo University of Science, 2641 Yamazaki, Noda, Chiba 278-8510, Japan; 3 Department of Biology, Tokyo Gakugei University, Koganei, Tokyo 184-8501, Japan; 4 United Graduate School of Agricultural Science, Tokyo University of Agriculture and Technology, Fuchu, Tokyo 183-8509, Japan; 5 Biomembrane Laboratory, Tokyo Metropolitan Institute of Medical Science, Setagaya-ku, Tokyo 156-8506, Japan; 6 Environmental Biology Division, National Institute for Environmental Studies, Tsukuba, Ibaraki, 305-8506, Japan; 7 RIKEN Plant Science Center, Tsukuba, Ibaraki 305-0074, Japan

## Abstract

**Background:**

Mechanosensing and its downstream responses are speculated to involve sensory complexes containing Ca^2+^-permeable mechanosensitive channels. On recognizing osmotic signals, plant cells initiate activation of a widespread signal transduction network that induces second messengers and triggers inducible defense responses. Characteristic early signaling events include Ca^2+ ^influx, protein phosphorylation and generation of reactive oxygen species (ROS). Pharmacological analyses show Ca^2+ ^influx mediated by mechanosensitive Ca^2+ ^channels to influence induction of osmotic signals, including ROS generation. However, molecular bases and regulatory mechanisms for early osmotic signaling events remain poorly elucidated.

**Results:**

We here identified and investigated OsMCA1, the sole rice homolog of putative Ca^2+^-permeable mechanosensitive channels in Arabidopsis (MCAs). OsMCA1 was specifically localized at the plasma membrane. A promoter-reporter assay suggested that *OsMCA1 *mRNA is widely expressed in seed embryos, proximal and apical regions of shoots, and mesophyll cells of leaves and roots in rice. Ca^2+ ^uptake was enhanced in *OsMCA1*-overexpressing suspension-cultured cells, suggesting that OsMCA1 is involved in Ca^2+ ^influx across the plasma membrane. Hypo-osmotic shock-induced ROS generation mediated by NADPH oxidases was also enhanced in *OsMCA1-*overexpressing cells. We also generated and characterized *OsMCA1-RNAi *transgenic plants and cultured cells; *OsMCA1-*suppressed plants showed retarded growth and shortened rachises, while *OsMCA1*-suppressed cells carrying Ca^2+^-sensitive photoprotein aequorin showed partially impaired changes in cytosolic free Ca^2+ ^concentration ([Ca^2+^]_cyt_) induced by hypo-osmotic shock and trinitrophenol, an activator of mechanosensitive channels.

**Conclusions:**

We have identified a sole MCA ortholog in the rice genome and developed both overexpression and suppression lines. Analyses of cultured cells with altered levels of this putative Ca^2+^-permeable mechanosensitive channel indicate that OsMCA1 is involved in regulation of plasma membrane Ca^2+ ^influx and ROS generation induced by hypo-osmotic stress in cultured rice cells. These findings shed light on our understanding of mechanical sensing pathways.

## Background

Plants need to sense and respond to mechanical stresses, such as wind, touch, and changes in osmotic pressure [1-3]. Elevation of cytosolic free Ca^2+ ^concentration ([Ca^2+^]_cyt_) is induced in response to various stimuli, such as chemical, physical, and mechanical stimuli [2,4-7]. During this process, [Ca^2+^]_cyt _levels rise through the opening of Ca^2+ ^channels located on the plasma membrane and endomembranes. Electrophysiological and bioinformatic studies have revealed the existence of plasma membrane Ca^2+^-permeable channels activated by mechanical stimuli, although the structural entity involved and their physiological functions remain largely unknown [8-12].

Molecular and electrophysiological studies have shown that *Arabidopsis thaliana *MSL9 and MSL10, homologs of the bacterial mechanosensitive channel MscS, are required for mechanosensitive channel activity in the plasma membrane of root cells, and are more permeable to Cl^- ^than Ca^2+ ^[13,14]. We have recently identified two plasma membrane proteins as putative Ca^2+^-permeable mechanosensitive channels, MCA1 (At4g35920) and MCA2 (At2g17780), from Arabidopsis [15,16], and showed that ectopic overexpression of *MCA1 *increases Ca^2+ ^uptake in roots, and also enhances [Ca^2+^]_cyt _elevation upon hypo-osmotic shock. However, the direct effects of MCA proteins on osmotic-induced Ca^2+ ^influx through the plasma membrane and the osmotic signaling pathways are little understood.

Upon recognition of osmotic signals, plant cells initiate activation of a widespread signal transduction network that induces second messengers and triggers inducible defense responses. Characteristic early signaling events include Ca^2+ ^influx, protein phosphorylation and generation of reactive oxygen species (ROS) [17-20]. These downstream events are often prevented when Ca^2+ ^influx is compromised by either Ca^2+ ^chelators, such as ethylene glycol-bis-(2-aminoethylether)-*N, N, N', N'*-tetra acetic acid (EGTA), or Ca^2+^-channel blockers, such as La^3+ ^[21]. In tobacco cells, hypo-osmotic shock-induced ROS generation reportedly requires activation of mechanosensitive Ca^2+ ^channels [22]. These results suggest that Ca^2+ ^influx mediated by mechanosensitive Ca^2+ ^channels is involved in the induction of osmotic signals including ROS generation. However, in osmotic responses, molecular bases and regulation mechanisms remain poorly elucidated.

In the present study, we have identified a sole MCA ortholog in the rice genome and developed both overexpression and suppression lines. Studies of these lines with altered levels of this putative mechanosensitive Ca^2+ ^channel indicated that OsMCA1 is involved in regulation of plasma membrane Ca^2+ ^influx and ROS generation induced by hypo-osmotic stress in cultured rice cells.

## Results

### Identification of OsMCA1 and its expression patterns

Full-length cDNA of *OsMCA1 *was obtained by a rapid amplification of cDNA ends (RACE)-PCR method. It encodes a polypeptide of 418 amino acid residues with a calculated molecular mass of 47,417 (GenBank Accession No. AB601973). The predicted protein showed 66.7% and 57.6% amino acid sequence identity compared with Arabidopsis MCA1 and MCA2, respectively; the TopPred program http://www.sbc.su.se/~erikw/toppred2/ suggests that OsMCA1 has two potential transmembrane segments (S1 and S2) (Additional file 1), while other transmembrane segment prediction programs suggest different numbers of putative transmembrane segments (data not shown). The PLAC8 motif was found by TMpred prediction http://www.ch.embnet.org/software/TMPRED_form.html in the C-terminal region (Additional file 1). A database search of the whole genome (Rice BLAST; http://riceblast.dna.affrc.go.jp/) indicated that rice has no other homolog of *OsMCA1*.

**Figure 1:**
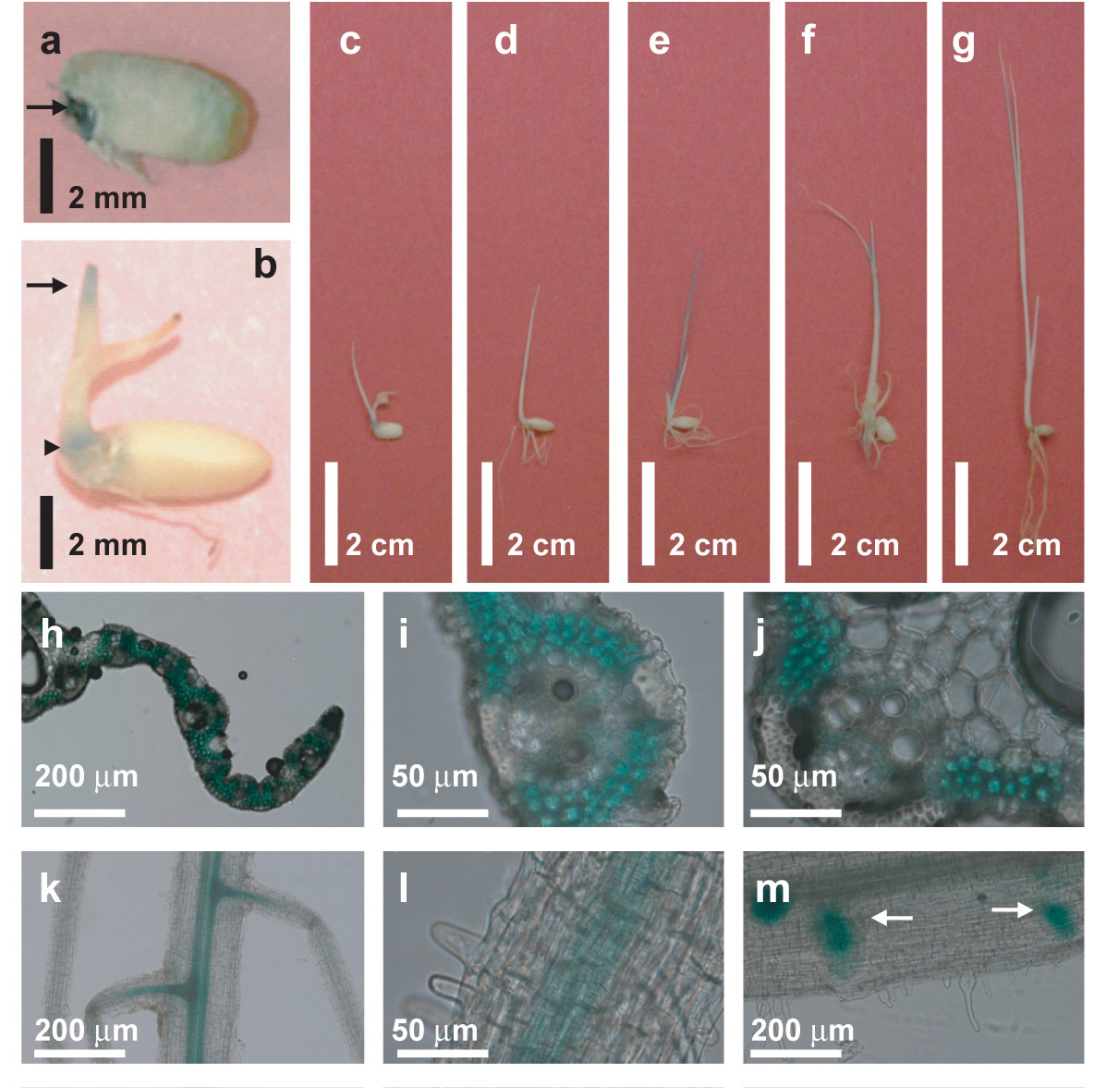
**Spatial patterns of *OsMCA1 *transcription as revealed by *GUS *staining**. Transgenic rice plants harboring *OsMCA1p::GUS *were stained in X-Gluc solution and cleared in methanol. (**a**) Half of a seed. An arrow indicates the seed embryo. (**b-g**) Whole seedlings grown for 7, 7, 7, 8, 13 and 11 days, respectively. Shoot apices and proximal regions are indicated with arrows and arrowheads, respectively. (**h-j**) Cross sections of leaves from plants grown for 14 days. Representative staining images for three transgenic plants are shown. (**k-m**) Roots from plants grown for 7, 14 and 14 days, respectively. Arrows indicate lateral root primordial. Sections are 50 or 200 μm thick.

Quantitative reverse transcriptase (RT)-PCR analysis showed *OsMCA1 *mRNA to be expressed in mature leaves, shoots, roots and suspension-cultured cells, suggesting that *OsMCA1 *mRNA is expressed throughout the plants in seedlings as well as in cultured cells (Additional file 2). We also consulted the microarray expression database (Rice XPro; http://ricexpro.dna.affrc.go.jp/GGEP/index.html, *OsMCA1 *locus ID; Os03g0157300), showing expression of *OsMCA1 *mRNA throughout the developmental stages, including root, leaf blade, panicle, anther, pistil, and ovary as well as embryo (data not shown). The spatial pattern of *OsMCA1 *expression was examined using an *OsMCA1 *promoter::*β*-glucuronidase (*GUS*) fusion reporter gene construct (*OsMCA1*p::*GUS*). Figure 1a shows *OsMCA1*p::*GUS *is expressed in the seed, with relatively high levels in embryo. In the seedling stage, *OsMCA1*p::*GUS *is highly expressed in proximal and apical regions of shoots (Figure 1b-g). Cross sections of the leaves indicate that *OsMCA1*p::*GUS *is highly expressed in mesophyll cells, but expressed in vascular tissues at relatively very low levels (Figure 1h-j). *OsMCA1*p::*GUS *was also expressed in the root, with relatively high levels in the center of primary root as well as the lateral root primordia (Figure 1k-m). These results suggest that *OsMCA1 *transcription may be regulated throughout developmental stages. The expression pattern of *OsMCA1 *was similar to those of the Arabidopsis *MCA2*.

### Intracellular localization of the OsMCA1 protein

**Figure 2:**
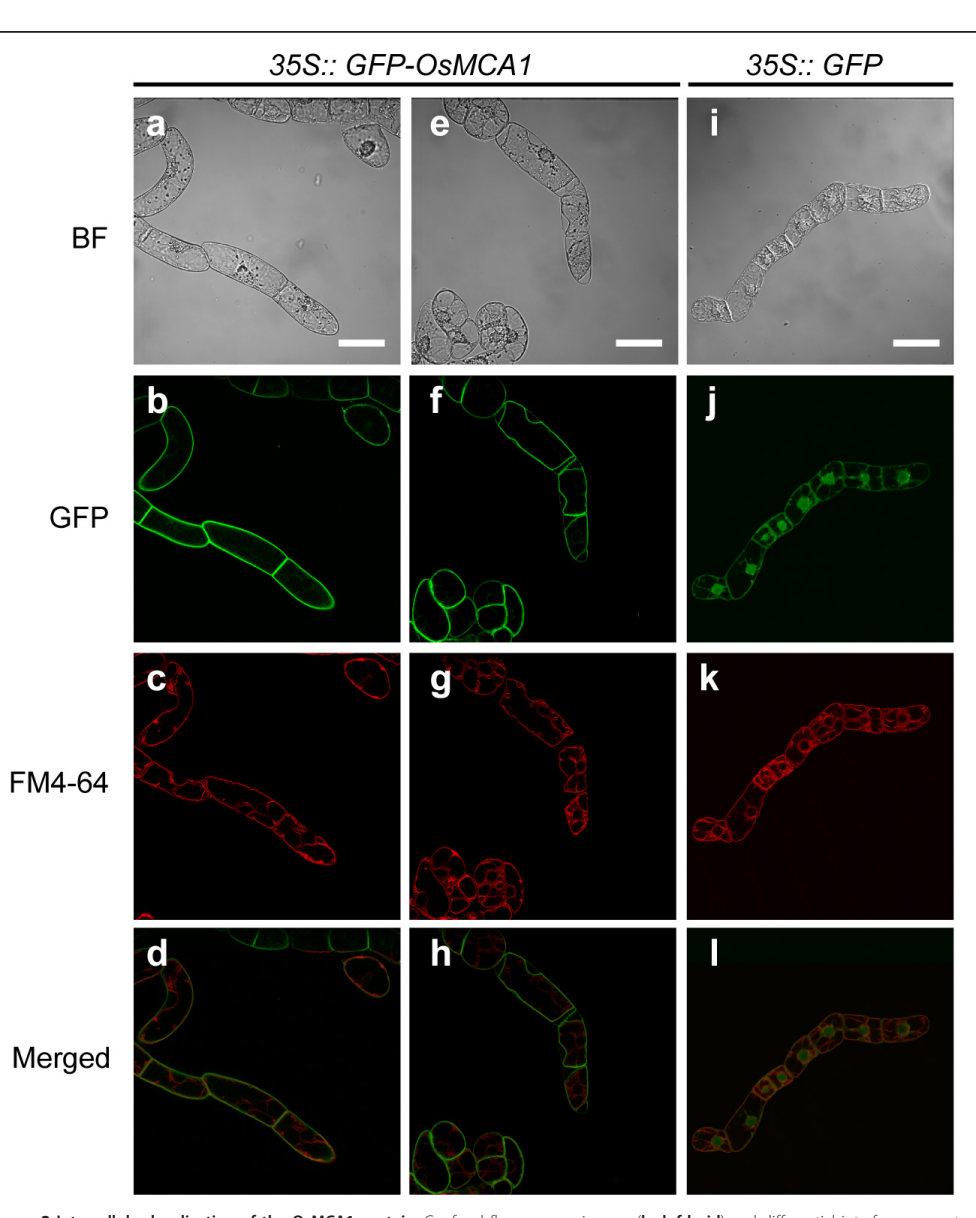
**Intracellular localization of the OsMCA1 protein**. Confocal fluorescence images (**b-d, f-h, j-l**) and differential interference contrast (DIC) images (**a, e, i**) of tobacco BY-2 cells expressing GFP-OsMCA1 (**a-h**) or GFP (**i-l**) stained with FM4-64 (4.25 μM) for 3 h. Fluorescence of GFP (**b, f, j**) and FM4-64 (**c, g, k**). (**e-h**) Plasmolyzed cells. Scale bar: 20 μm.

To investigate intracellular localization of the OsMCA1 protein, we introduced a green fluorescent protein (GFP) construct fused to the coding sequence of the N-terminus of OsMCA1 into tobacco BY-2 cells and examined its intracellular localization using confocal laser scanning microscopy. When GFP alone was expressed, it localized to the nucleus and the cytoplasm (Figure 2i, j). In contrast, GFP-OsMCA1 fusion protein localized specifically to the plasma membrane (Figure 2a, b). This pattern was reinforced by treatment with a high osmotic solution, 1 M mannitol, which induced plasmolysis (Figure 2e, f). In addition, fluorescent images and behavior of GFP-OsMCA1 before and after plasmolysis were different from those of the intracellular staining marker FM4-64 (Figure 2c, g, k and 2d, h, l), indicating that OsMCA1 is localized to the plasma membrane.

### Effects of OsMCA1 overexpression on Ca^2+ ^uptake in cultured rice cells

**Figure 3:**
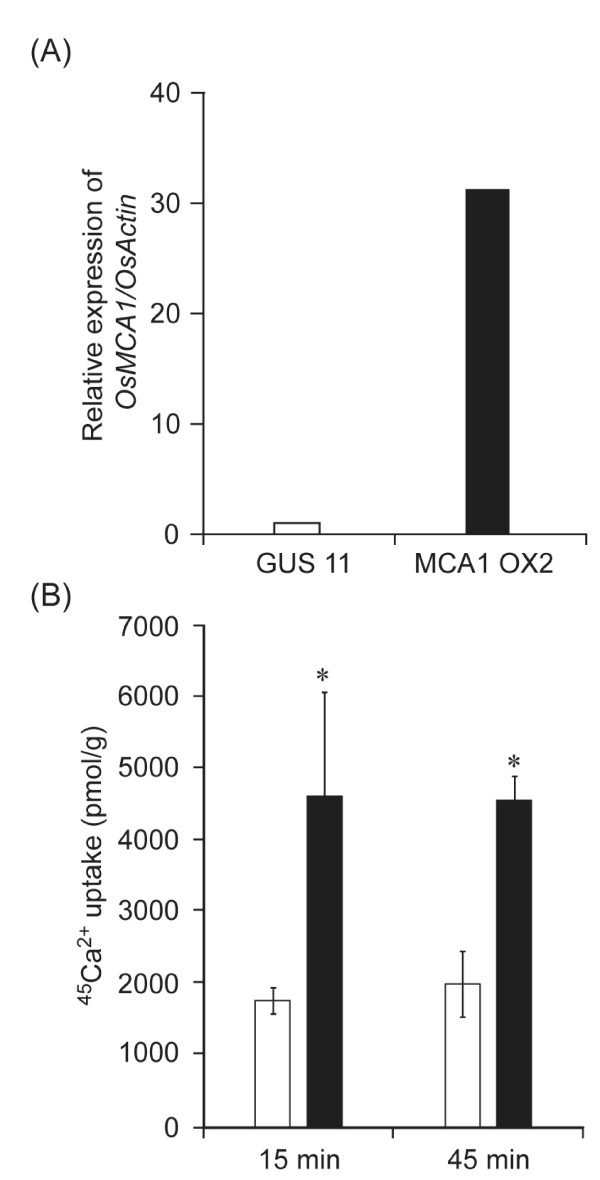
**Effect of *OsMCA1*-overexpression on Ca^2+ ^uptake in cultured rice cells**. Open and closed bars represent *GUS*-expressing control line (GUS No.11) and *OsMCA1-*overexpressing line (OX No. 2), respectively. (A) Quantification of *OsMCA1 *mRNA by real-time quantitative PCR. The amount of *OsMCA1 *mRNA was calculated from the threshold point in the log-linear range of the RT-PCR. The relative level of *OsMCA1 *mRNAs in the *GUS*-expressing control line was standardized as 1. (B) ^45^Ca^2+ ^uptake into cultured rice cells. Data are means ± SD, n = 3 independent samples. *P < 0.05; significantly different compared with the control.

To test if OsMCA1 plays a role in Ca^2+ ^transport, we generated cultured cells overexpressing *OsMCA1 *and analyzed whether expression levels of *OsMCA1 *affected Ca^2+ ^uptake activity. As shown in Figure 3A, B, Ca^2+ ^uptake activity was higher in *OsMCA1*-overexpressing cells than in *GUS*-expressing control cells, suggesting that OsMCA1 is involved in Ca^2+ ^uptake across the plasma membrane in rice. We also generated *OsMCA1*-overexpressing plants, which showed no significant visible phenotypes (data not shown).

### Effect of OsMCA1 suppression on growth and development in planta

**Figure 4:**
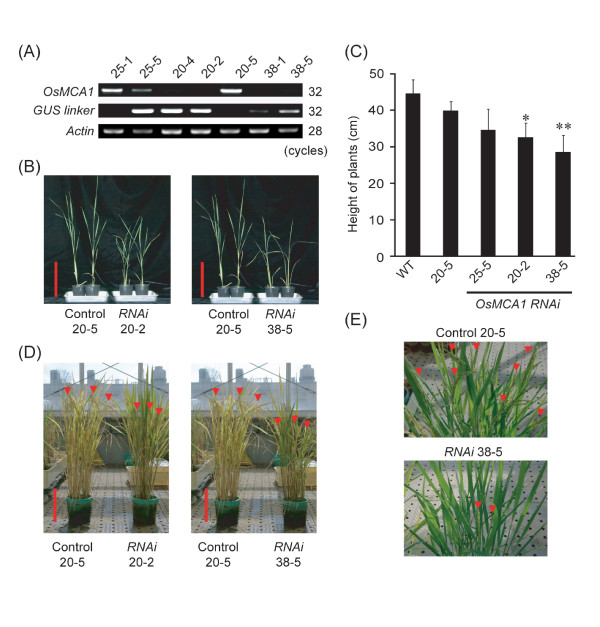
**Effects of *OsMCA1 *suppression on growth and development *in planta***. (**A**) RT-PCR analysis of *OsMCA1 *in five independent *RNAi *lines. Expression of *GUS *linker indicates RT-PCR products of *GUS *linker region, and expression of trigger dsRNA. *Actin *cDNA was used as internal control; PCR products were analyzed by agarose gel electrophoresis. (**B**) Phenotype of *OsMCA1*-suppressed lines of 3-week-old plants. Bars indicate 10 cm. (**C**) Heights of plants shown in (**B**) were quantified. Data are means ± SD, n = 6-13 independent plants. *P < 0.05; **P < 0.01; significantly different compared with two control lines (WT and Control 20-5). (**D, E**) Phenotype of *OsMCA1*-suppressed lines grown for 4 months (D) and 100 days (E) in a greenhouse. Arrowheads indicate ears; bars indicate 10 cm.

To elucidate the physiological roles of OsMCA1, transgenic plants were generated in which *OsMCA1 *expression was suppressed by RNA interference (*RNAi*) using gene-specific sequences (a 400-bp region of *OsMCA1*). Five independent transgenic plants were generated using *Agrobacterium*-mediated transformation. Non-transgenic plants were investigated simultaneously as controls, whose transduced genes were removed by heterozygous segregation. RT-PCR analyses revealed significant reductions in *OsMCA1 *mRNA levels compared with controls (Figure 4A).

The *OsMCA1*-suppressed lines showed slower growth in adult plants (Figure 4B, C). Though germination rates (data not shown) and seedling growth of suppression lines were comparable to controls in the Murashige and Skoog medium (MS medium) (Additional file 3), growth of suppression lines was remarkably retarded after transplantation into soil in a greenhouse, suggesting that *OsMCA1 *suppression leads to hypersensitivity to environmental stresses. This phenotype was exhibited in all 5 independent T_2 _transgenic *RNAi *lines; severity of the phenotypes correlated well with expression levels of *OsMCA1 *transcripts (Figure 4A, C). Furthermore, unlike Arabidopsis *mca *mutants, rachises of the *OsMCA1-*suppressed lines were significantly shorter than those of controls (Figure 4D, E), suggesting that OsMCA1 plays a different role from Arabidopsis MCAs in some developmental stages.

### Effects of OsMCA1-suppression on cell growth and Ca^2+ ^sensitivity in cultured rice cells

**Figure 5:**
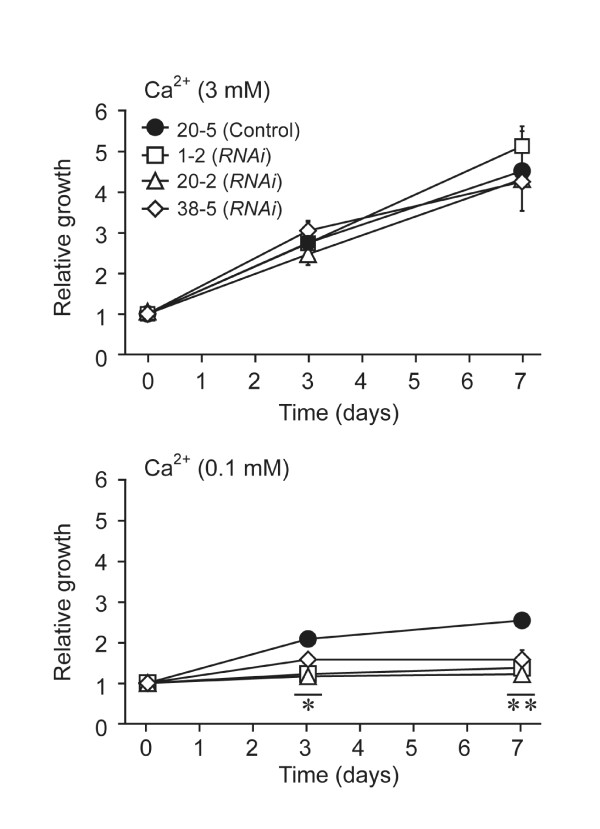
**Effects of *OsMCA1 *suppression on cell growth and Ca^2+ ^sensitivity in cultured rice cells**. Seven day-old-cultured cells were assayed. Cells (fresh weight 0.5 g) were transferred to L medium with standard or low Ca^2+ ^concentration. After culturing for 0, 3, or 7 days, the fresh weight of cells was measured. Data are the mean ± SE for three independent experiments. * *P *< 0.05; ** *P *< 0.01; significantly different compared with control.

We tested whether *OsMCA1 *suppression affects Ca^2+ ^sensitivity to growth in rice cells. In regular medium containing 3 mM of Ca^2+^, growth rates of *OsMCA1*-suppressed lines were comparable to controls (Figure 5). In contrast, when Ca^2+ ^concentration of the medium was decreased to 0.1 mM (Figure 5), growth of *OsMCA1*-suppressed lines was significantly restricted compared with controls, suggesting possible involvement of OsMCA1 in acquisition of Ca^2+ ^for cell growth under Ca^2+ ^limitation.

### Involvement of OsMCA1 in mechanical stress-induced [Ca^2+^]_cyt _changes

**Figure 6:**
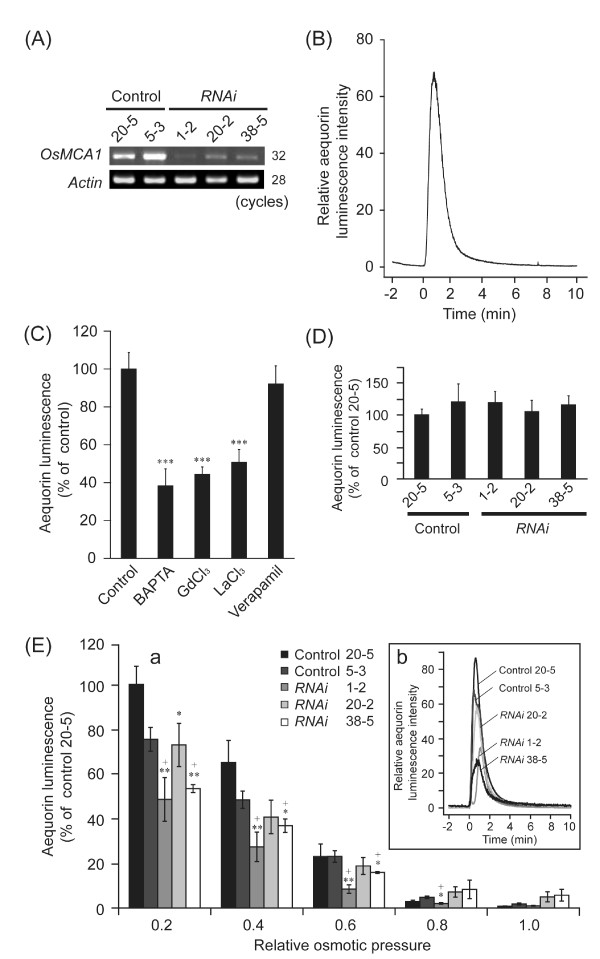
**Involvement of OsMCA1 in the regulation of hypo-osmotic shock-induced [Ca^2+^]_cyt _changes**. (**A**) RT-PCR analysis of *OsMCA1 *in three independent *RNAi *lines expressing *apoaequorin *mRNA. (**B**) Hypo-osmotic shock-induced [Ca^2+^]_cyt _changes in cultured rice cells. Cells were diluted four-fold with water at 0 min. A representative result of five experiments is shown. (**C**) Pharmacological analyses of hypo-osmotic shock-induced [Ca^2+^]_cyt _changes. Cells were diluted four-fold with water at 0 min. BAPTA (5 mM), GdCl_3 _(5 mM), LaCl_3 _(5 mM) and verapamil (1 mM) were added to the cell suspension 15 min before hypo-osmotic shock treatment. No treatment prior to hypo-osmotic shock in the "control." Peak intensities of aequorin chemiluminescence were compared; relative luminescence level in the control was standardized as 100% (**C **and **D**). Data are mean ± SE for five independent experiments. ***P < 0.005; significantly different compared with control. (**D**) Effects of *OsMCA1 *suppression on *N*-acetylchito-oligosaccharides-induced [Ca^2+^]_cyt _changes. Cells were treated with *N*-acetylchitoheptaose (1 μM). Error bars indicate SE of the mean of five experiments. (E-a) 1 mL of water, medium, or diluted medium was added to *OsMCA1*-suppressed cells harboring apoaequorin at 0 min to generate wide-ranging changes in extracellular osmotic pressure. The relative amount of luminescence accumulated for 10 min after hypo-osmotic shock was plotted versus relative extracellular osmotic pressure. Data are means ± SE, n = 3-7. ^+^*P < 0.05; ^+^**P < 0.01; significantly different compared with two control lines (No. 20-5 and 5-3). *P < 0.05; significantly different compared with the control line (No. 20-5). (E-b) Hypo-osmotic shock-induced Ca^2+ ^signature in *OsMCA1*-suppressed lines. Cells were diluted four-fold with water at 0 min. A representative result of several experiments is shown.

To test for possible involvement of OsMCA1 in regulation of Ca^2+ ^influx induced by various stimuli, we generated *OsMCA1*-suppressed lines harboring the Ca^2+^-sensitive bioluminescent protein aequorin (Figure 6A). Hypo-osmotic shock-induced transient [Ca^2+^]_cyt _change in cultured rice cells (Figure 6B) was inhibited by a Ca^2+ ^chelator, 1,2-bis-(2-aminophenoxy)ethane-*N, N, N', N'*-tetra-acetic acid (BAPTA), and Ca^2+ ^channel blockers (GdCl_3 _and LaCl_3_) but not by verapamil, an inhibitor for voltage-dependent Ca^2+ ^channels, (Figure 6C), suggesting that plasma membrane Ca^2+ ^influx mediated by Gd^3+^-sensitive mechanosensitive Ca^2+^-permeable channel(s) is induced by hypo-osmotic shock. The hypo-osmotic shock-induced [Ca^2+^]_cyt _change was partially impaired in the *OsMCA1*-suppressed cells (Figure 6E), and was proportional to levels of *OsMCA1 *expression in various *OsMCA1*-suppressed cells. On the other hand, increased [Ca^2+^]_cyt _triggered by *N*acetylchito-oligosaccharides, a major microbe-associated molecular pattern (MAMP) recognized by plasma membrane receptors in rice [23,24], was not affected by *OsMCA1 *expression levels (Figure 6D). These results suggest that OsMCA1 participates in the plasma membrane Ca^2+ ^influx triggered by hypo-osmotic shock but not by MAMPs.

**Figure 7:**
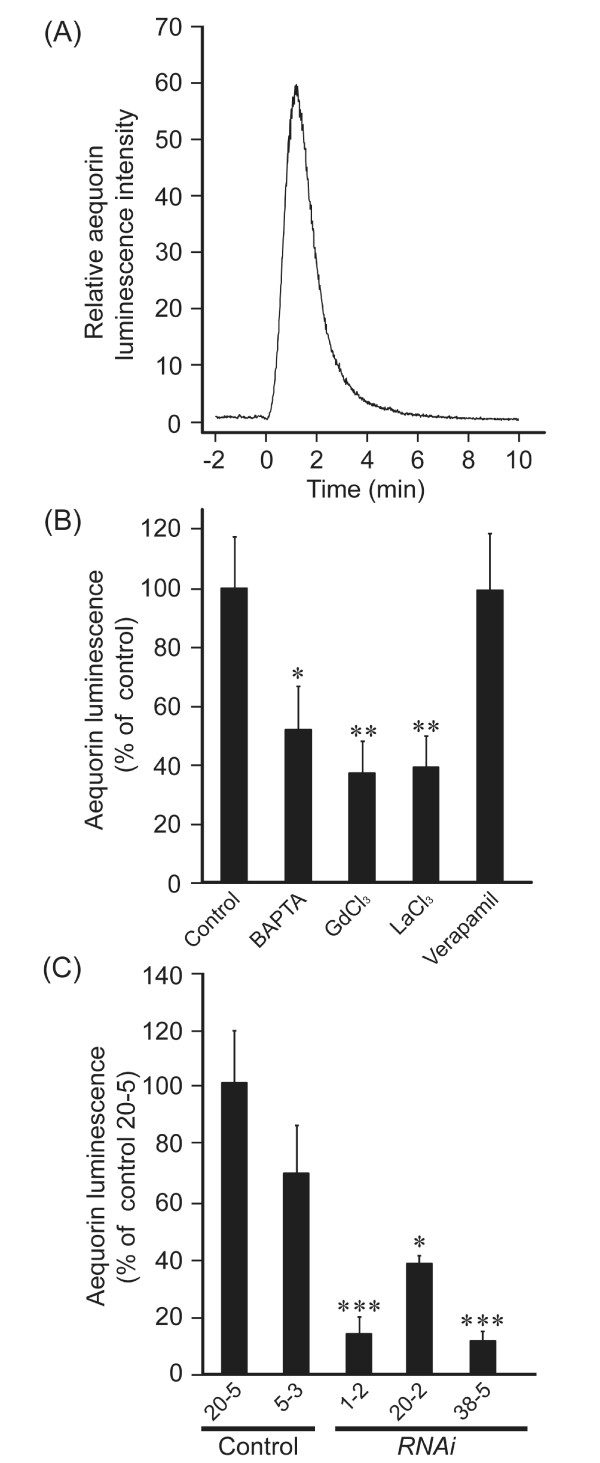
**Involvement of OsMCA1 in the regulation of Trinitrophenol (TNP)-induced [Ca^2+^]_cyt _changes**. (**A**) TNP-induced [Ca^2+^]_cyt _changes in cultured rice cells. Cells were treated with TNP (1 mM) at 0 min. A representative result of five experiments is shown. (**B**) Pharmacological analyses of TNP-induced [Ca^2+^]_cyt _changes. BAPTA (5 mM), GdCl_3 _(5 mM), LaCl_3 _(5 mM) and verapamil (1 mM) were added to the cell suspension 15 min prior to the TNP treatment; DMSO is used as the control. The peak intensities of aequorin chemiluminescence were compared; the relative luminescence level in the control was standardized as 100%. Data are the mean ± SE for three independent experiments. *P < 0.05; **P < 0.01; significantly different compared with control. (**C**) Effects of *OsMCA1*-suppression on TNP-induced [Ca^2+^]_cyt _changes. Cells were treated with TNP (1 μM). The peak intensities of aequorin chemiluminescence were compared; the relative luminescence level in the control line (20-5) was standardized as 100%. Data are the mean ± SE for three independent experiments. *P < 0.05; ***P < 0.005; significantly different compared with two control lines (20-5 and 5-3).

Trinitrophenol (TNP) is a potent compound to generate membrane distortion to activate mechanosensitive channels and mimic mechanical stimuli in plants [15] and animal cells. We investigated the effects of TNP on [Ca^2+^]_cyt _and possible involvement of OsMCA1 in its regulation in cultured rice cells. TNP induced transient [Ca^2+^]_cyt _change, which was inhibited by BAPTA, GdCl_3_, and LaCl_3 _but not by verapamil (Figure 7A, B). The pharmacology of [Ca^2+^]_cyt _transients triggered by hypo-osmotic shock and TNP was basically similar (Figure 6C, 7B). TNP-induced [Ca^2+^]_cyt _change was also impaired in *OsMCA1*-suppressed lines (Figure 7C), suggesting the possible involvement of OsMCA1 as a putative mechanosensitive Ca^2+^-permeable channel in the regulation of mechanical stress-triggered plasma membrane Ca^2+ ^influx.

We also tried to examine the effect of overexpression of *OsMCA1 *on mechanical stress-triggered Ca^2+ ^influx. However, we observed a strong reduction in the total aequorin luminescence in all transgenic cell lines overexpressing *OsMCA1 *(data not shown). Thus it was impossible to measure [Ca^2+^]_cyt _using *OsMCA1*-overexpressing lines. Real-time RT-PCR analysis revealed that the expression level of aequorin gene in the *OsMCA1*-overexpressing lines was comparable to the control (data not shown). Thus constitutive overexpression of *OsMCA1 *does not affect the expression but may affect the stability of aequorin protein or inhibit the aequorin chemiluminescence.

### Effects of OsMCA1-overexpression on sensitivity to hypo-osmotic shock and generation of reactive oxygen species

Hypo-osmotic shock has been shown to trigger ROS generation following [Ca^2+^]_cyt _increase in cultured tobacco cells [18,22]. As OsMCA1 has been suggested to affect regulation of hypo-osmotic shock-induced Ca^2+ ^influx (Figure 6E), we investigated possible OsMCA1 involvement in hypo-osmotic shock-induced ROS generation in cultured rice cells, using two distinctive methods sensitive for superoxide anion radical (^•^O_2_^-^) and hydrogen peroxide (H_2_O_2_).

**Figure 8:**
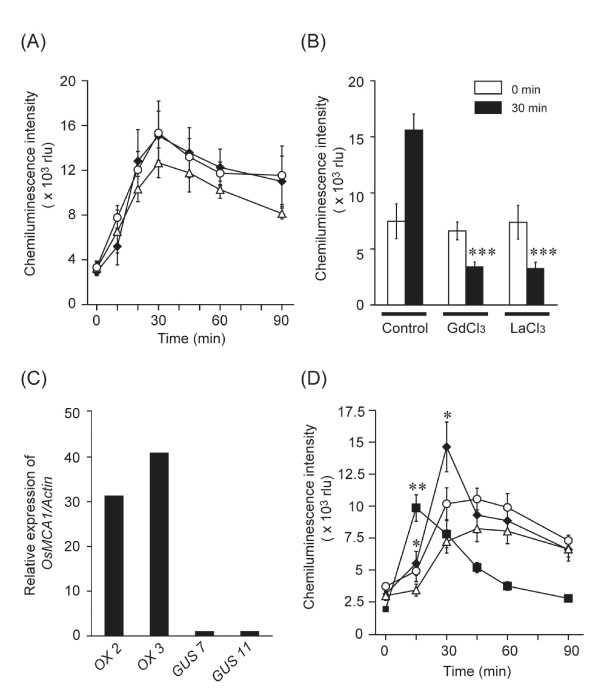
**Effects of the expression level of *OsMCA1 *on hypo-osmotic shock-induced ROS generation**. (**A**) Effects of *OsMCA1*-suppression on hypo-osmotic shock-induced ROS generation measured by MCLA chemiluminescence. Data are the mean ± SE for five independent experiments for the control line (closed diamond for control 20-5) and two independent *RNAi *lines (open circle for *RNAi *20-2; open triangle for *RNAi *38-5) are shown. As a hypo-osmotic shock, growth medium was replaced by four-fold diluted medium at 0 min (**A **and **B**). (**B**) The effect of several Ca^2+ ^channel blockers on hypo-osmotic shock-induced ROS generation in the wild type. GdCl_3 _(1 mM) and LaCl_3 _(1 mM) were added to the cells 60 min prior to hypo-osmotic shock treatment. Data are the mean ± SE for three or four independent experiments. ***P < 0.005; significantly different compared with the control. (**C**) Quantitative expression levels of *OsMCA1 *mRNAs in the *OsMCA1 *overexpressor lines by real-time quantitative PCR. The relative level of the *OsMCA1 *mRNAs in the control cells (GUS No. 7) was standardized as 1. (**D**) Effects of *OsMCA1 *overexpression on hypo-osmotic shock-induced ROS generation. As a hypo-osmotic shock, growth medium was replaced by three-fold diluted medium at 0 min. Data are the mean ± SE for five independent experiments for two control lines (open circle for GUS No. 11; open triangle for GUS No. 7) and two overexpressor lines (closed diamond for OX No. 2; closed square for OX No. 3) are shown. *P < 0.05; **P < 0.01; significantly different compared with the control line (GUS No. 7).

Hypo-osmotic shock triggered ROS generation within 5 min (Figure 8A), which was markedly inhibited by Ca^2+ ^channel blockers (GdCl_3 _and LaCl_3_), suggesting an important role f Ca^2+ ^influx in hypo-osmotic shock-induced ROS generation (Figure 8B). Diphenylene iodonium (DPI; 10 μM), an NADPH oxidase inhibitor, significantly suppressed ROS generation (Additional file 4). Peroxidase-catalyzed reactions have been also proposed for osmotic shock-induced ROS generation in cultured tobacco and *Arabidopsis *cells [19]. However, a peroxidase inhibitor, salicylhydroxamic acid (SHAM, 3 mM), scarcely affected hypo-osmotic shock-induced ROS generation (Additional file 5), suggesting that a major part of hypo-osmotic shock-induced ROS generation is attributable to ROS-producing NADPH oxidases in cultured rice cells. Hypo-osmotic shock-induced generation of both ^•^O_2_^- ^(Figure 8C, D) and H_2_O_2 _(Additional file 6) was either significantly enhanced or more rapid in *OsMCA1*-overexpressing lines than in the control. Generation of ROS in *OsMCA1*-suppressed lines was comparable to the control (Figure 8A).

## Discussion

It has been suggested that Ca^2+ ^plays a crucial role in mechanical sensing [7,25]. However, little is known of the molecular mechanisms responsible for Ca^2+ ^mobilization. Functional characterization of the *OsMCA1-RNAi *lines as well as overexpressors suggests that OsMCA1 is involved in hypo-osmotic shock-induced Ca^2+ ^influx and ROS generation.

### Possible functions of OsMCA1 in the regulation of growth and development of rice

*OsMCA1*-suppressed plants displayed stunted growth and shortened rachises (Figure 4D, E). These phenotypes are frequently observed under drought-stress conditions [26]. The suppression of *OsMCA1 *might have affected adaptation to drought. Drought stress is known to lead to osmotic stress at a cellular level. Since hypo-osmotic shock-induced [Ca^2+^]_cyt _changes were impaired in the *OsMCA1*-suppressed lines, these lines may have defects in osmotic sensing/responses and ability to adapt to drought stress. Future studies to characterize physiological reactions to drought and mechanical signaling in *OsMCA1-*suppressed plants would further elucidate the in vivo roles of OsMCA1 in intact plants.

In Arabidopsis, the *mca1 mca2 *double mutant shows a growth defect in soil, and reduced accumulation of Ca^2+ ^as well as enhanced sensitivity to Mg^2+ ^[16]. The balance of Ca^2+ ^and Mg^2+ ^in soil is an important factor for normal plant growth [27]. Since the growth of the *OsMCA1*-suppressed lines was significantly restricted compared with the control under Ca^2+^-limitation (Figure 5), growth retardation in the *OsMCA1*-suppressed plants may be attributed to reduced Ca^2+ ^uptake, resulting in a low Ca^2+^-Mg^2+ ^ratio.

### Possible involvement of OsMCA1 in osmotic signaling in cultured rice cells

The GFP-OsMCA1 fusion protein localized specifically to the plasma membrane (Figure 2), suggesting that OsMCA1 is a plasma membrane protein. In cultured rice cells, both hypo-osmotic shock- and TNP-induced [Ca^2+^]_cyt _transients, which were inhibited by La^3+ ^and Gd^3+^, were impaired in *OsMCA1*-suppressed lines (Figures 6 and 7). The temporal pattern of the MAMP-induced [Ca^2+^]_cyt _transient was similar (data not shown), but was unaffected by *OsMCA1 *suppression (Figure 6D). These results suggest that OsMCA1 affects regulation of Ca^2+ ^influx across the plasma membrane in response to mechanical stimulation in cultured rice cells.

Hypo-osmotic shock triggers ROS generation following a [Ca^2+^]_cyt _increase [18,20,22]. Extracellular Ca^2+ ^is required for both Ca^2+ ^influx and NADPH oxidase-mediated ROS generation induced by hypo-osmotic shock (Figures 6C and 8B, Additional file 4), suggesting that ROS generation requires Ca^2+ ^influx across the plasma membrane. Overexpression of *OsMCA1 *enhances ROS generation (Figure 8D, Additional file 6). Binding Ca^2+ ^to the EF-hand regions of cytosolic regulatory domains of plant NADPH oxidases directly activates them [28-30]. A functional NADPH oxidase AtrbohC/RHD2 reportedly affects mechanical stress-induced ROS generation in a Ca^2+^-dependent manner [31]. Overproduction of the plasma membrane Ca^2+^-permeable channels may induce the mobilization of excess Ca^2+ ^in response to mechanical stimuli, which may cause enhanced activation of NADPH oxidases.

In *OsMCA1*-suppressed lines challenged with hypo-osmotic shock, Ca^2+ ^influx was partially impaired (Figure 6E), but no significant influence of the impairment on subsequent ROS generation was detected under our assay conditions (Figure 8A). Similar effects of overexpression and a loss-of-function mutation were also observed with another putative Ca^2+^-permeable channel, OsTPC1 [32]. A certain level of Ca^2+ ^increment may be sufficient for NADPH oxidase-mediated ROS generation. Alternatively, other Ca^2+^-permeable channels activated by hypo-osmotic shock may redundantly play a role in bypassing OsMCA1. It has been suggested that Arabidopsis MSL9 and MSL10, homologs of the bacterial mechanosensitive channel MscS, are required for mechanosensitive channel activity in root cell plasma membranes, and are able to translocate cations including Ca^2+ ^[13,14]. Rice MSL homologs may therefore be candidates for such Ca^2+^-permeable channels.

## Conclusions

The present study indicates that OsMCA1 is involved in regulation of plasma membrane Ca^2+ ^influx and NADPH oxidase-mediated ROS generation induced by hypo-osmotic stress in cultured rice cells. These findings shed light on our understanding of mechanical sensing pathways.

## Methods

### Plant materials and cell culture

Surface-sterilized seeds of rice, *Oryza sativa L*. cv. Nipponbare, were germinated on MS medium [33] containing 0.8% agar and grown for 10 days in a growth chamber under long day conditions (16 h light/8 h darkness, 28°C). Seedlings were transplanted into soil and grown in a greenhouse (16 h light/8 h darkness, 28°C and 60% humidity). Calli were suspension-cultured at 25°C in a liquid L medium [34] containing 2,4-D (0.5 mg L^-1^) in the dark and subcultured in fresh medium every week. Cells were filtered through a 20-mesh screen every 2 weeks to make fine aggregates. Cells at 5 days after subculture were used for experiments with osmotic stress and defense responses. *N*-acetylchito-oligosaccharides were kindly provided by Prof. Naoto Shibuya (Meiji University).

### Isolation of OsMCA1 cDNA

The estimated coding region of *OsMCA1 *was amplified by PCR using two primers: *OsMCA1 *forward, 5'-GAAGAAGAAGAAGAAGAAGAAGCCGAGTAG-3'; *OsMCA1 *reverse, 5'-TATTTATGCTTACCCTGCATTGTTTGTGTT-3'. Total RNA was isolated from rice leaves using Trizol (Invitrogen, Carlsbad, CA, USA) in accordance with manufacturer's protocol and quantified spectrophotometrically. First-strand cDNA was synthesized from 3 μg of total RNA with the oligo-dT primer and reverse transcriptase (Invitrogen). To obtain full-length cDNAs for *OsMCA1 *and to define the open reading frame, 3'-RACE PCR and 5'-RACE PCR were performed with a 3'-full RACE core kit (Takara, Ohtsu, Japan) and a 5'-RACE system (Invitrogen) in accordance with manufacturers' protocols.

### RNA isolation and RT-PCR analyses

Total RNA was isolated using Trizol reagent in accordance with manufacturer's protocol and quantified using a spectrophotometer. First-strand cDNA was synthesized from 3 μg total RNA with an oligo-dT primer and reverse transcriptase. PCR amplification was performed with an initial denaturation at 95°C for 3 min followed by indicated numbers of cycles of incubations at 94°C for 30 s, 55°C for 90 s, and 72°C for 1 min by using specific primers for *OsMCA1. Actin *was used as a quantitative control [35]. Aliquots of individual PCR products were resolved by agarose gel electrophoresis and visualized using ethidium bromide staining and exposure to UV light.

### Real-time RT-PCR quantification

Real-time RT-PCR assays were performed as described by Kurusu et al. (2010) [36]. First-strand cDNA was synthesized from 3 μg of total RNA using an oligo-dT primer and reverse transcriptase. Real-time PCR was performed using an ABI PRISM 7300 sequence detection system (Applied Biosystems, Foster City, CA, USA) with SYBR Green real-time PCR Master Mix (Toyobo, Osaka, Japan) and *OsMCA1 *specific primers OsMCA1-RealF, 5'-TGGTCTCAAGCAGAGGATCATACA-3'; OsMCA1-RealR, 5'-CTCTGAACAGCAACCAAGCAAA-3'. Relative mRNA levels were calculated using the standard curve method and normalized to the corresponding *OsActin1 *gene levels. Standard samples of known template amounts were used to quantify PCR products.

### Spatial pattern of OsMCA1 expression using OsMCA1p::GUS-expressing plants

A DNA fragment of the *OsMCA1 *promoter region was prepared using PCR by synthesizing the 5'-non-coding region spanning ^-^1.5 to 0 kb from the *OsMCA1 *initiation codon, using rice (Nipponbare) genomic DNA as a template and the following primers: OsMCA1pF, 5'-CACCAACAACCCCTAACATGCCTAA-3'; OsMCA1pR, 5'-TGCCGTCGTCTACTCGGCTTCTTCT-3' (the CACC sequence used with the Gateway system), subcloned into a pENTR/D-TOPO cloning vector, and then cloned into a Ti-based promoterless *GUS *expression vector, pHGWFS7 [37] using the LR clonase reaction; *Agrobacterium*-mediated transformation of rice calli was performed. Transformed calli were screened by hygromycin selection (50 μg mL^-1^); transgenic plants were then regenerated.

The T_2 _transgenic plants were grown at 28°C under a 16 h light/8 h dark cycle for experiments. Histochemical localization of GUS activity in situ was performed as follows. Samples were fixed for 1 h with 90% acetone in Eppendorf tubes placed on ice and washed four times with 100 mM sodium phosphate buffer, pH 7.0. Samples were then incubated for 24 h at 37°C in X-Gluc buffer (0.5 mg/mL 5-bromo-4-chloro-3-indolyl glucuronidase (Nacalai Tesque, Osaka, Japan), 50 mM sodium phosphate buffer, pH 7.0, 5% methanol), and then cleaned and fixed by rinsing for 1 h each with 50%, 70%, 90%, and 100% (v/v) ethanol successively. Fixed samples were stored in 100% ethanol before being photographed.

### Generation of OsMCA1-overexpressing and suppressed lines

To generate RNA-silencing-triggered inverted repeat constructs, a region corresponding to 400 bp of the 3'-UTR of *OsMCA1 *was amplified using RNAiFW, 5'-CACC CTCTTATCCAAACTTGCCAT-3' and RNAiRV, 5'- AATGTTCCACAGGGGAAAAAGAATGTTCTC-3' as specific primers, subcloned into a pENTR/D-TOPO cloning vector, and cloned into a Ti-based RNAi vector, pANDA [38] using the LR clonase reaction. The construct was introduced into rice calli using *Agrobacterium*-mediated transformation, according to the method of Tanaka et al. 2001 [39]. Transformed calli were screened by hygromycin selection (50 μg mL^-1^); transgenic plants were then regenerated. Transgenic cell lines derived from T_2 _plants were used for various analyses.

To overexpress *OsMCA1 *and *GUS *cDNAs, sequences were cloned into a Ti-based vector pPZP2H-lac [40] downstream of the maize *Ubiquitin *promoter, and *Agrobacterium*-mediated transformation of rice calli was performed. Transformed calli were screened by hygromycin selection (50 μg mL^-1^), followed by the regeneration of transgenic plants.

To express cytoplasm-targeted *apoaequorin *cDNA [41] in *OsMCA1*-suppressed plants, sequences were cloned into a Ti-based vector pSMAB704 [42] downstream of a *CaMV 35S* promoter, and *Agrobacterium*-mediated transformation of rice calli was performed. Transformed calli were screened using bialaphos (Meiji Seika, Tokyo, Japan) selection (5 μg mL^-1^), followed by the regeneration of transgenic plants.

### Subcellular localization of OsMCA1 in tobacco BY-2 cells

To generate transgenic BY-2 cells expressing GFP-OsMCA1, the coding region was amplified using OsMCA1(GFP)F, 5'-CACCATGGCGTCGTGGGAGAACCT-3' and OsMCA1(GFP)R, 5'-TTAGTGTTCCATGTACTGAA-3' as specific primers, subcloned into a pENTR/D-TOPO cloning vector, and then cloned into a pH7WGF2 vector encoding a N-terminal EGFP fusion [37] using the LR clonase reaction.

Transformation of BY-2 cells was carried out in accordance with An (1985) [43] with minor modifications as follows: 4 mL of 3-day-old exponentially growing culture was transferred to 90-mm Petri dishes and incubated at 28°C with 100 μL of fresh overnight-culture of *Agrobacterium tumefaciens *pGV2260 containing the binary vector pH7WGF2. After a 48-h co-cultivation, the tobacco cells were washed and plated on to LS agar medium containing hygromycin (50 μg mL^-1^) and carbenicillin (250 μg mL^-1^). Every 3-4 weeks, transformants were selected and transferred onto fresh medium for continued selection.

The fluorescent styryl membrane probe FM4-64 (Molecular Probes, Carlsbad, CA, USA) was kept as a 17 mM stock solution in sterile water, and used at a final concentration of 4.25 μM to label the vacuolar membrane (tonoplast). Five-day-old BY-2 cells were treated with FM4-64 for 3 h and washed twice with culture medium.

### Measurement of cytosolic Ca^2+ ^concentration

Measurements of Ca^2+ ^mobilization were made in accordance with the method described by Kurusu et al. (2011) [44]. Briefly, apoaequorin-expressing rice cells (5 day after subculture) were incubated with 1 μM coelenterazine for at least 12 h at 25°C. Cell suspension (250 μL) was transferred to 1.1-cm-diameter culture tubes, and set in a luminometer (Lumicounter 2500, Microtech Nition, Chiba, Japan). In the luminometer, culture tubes rotated 17 revolutions every 3 s clockwise and counterclockwise in turn, agitating the cells. After a 15-min incubation to stabilize the cells, Ca^2+^-dependent aequorin luminescence was measured and expressed as relative luminescence units (rlu).

### Ca^2+ ^uptake in cultured cells

Rice cells 5 days after subculture were used to measure Ca^2+ ^uptake. The rice cells were incubated in Ca^2+^-free medium for at least 3 h at 25°C. The cell suspension (80 mg mL^-1^) was transferred to medium containing 0.1 mM CaCl_2 _and incubated for 1 h. Ca^2+ ^uptake was initiated by adding ^45^CaCl_2 _solution to a final concentration of 33 kBq/g. Cells were then agitated at 25°C; 1 mL of cells was collected at 0, 15 and 45 min after the addition of ^45^CaCl_2_. Cells were filtered using Whatman filters (GF/C) presoaked with 5 mM CaCl_2 _and washed 5 times with an ice-cold solution of 5 mM CaCl_2_, and 2 mM LaCl_3 _to remove ^45^Ca^2+ ^from cell walls. Radioactivity retained on each filter was counted as described previously [45].

### Measurement of ROS

Rice cells (cv. Nipponbare) 5 days after subculture were used for measurement of ^•^O_2_^- ^and H_2_O_2 _in the extracellular medium. ^•^O_2_^-^-dependent chemiluminescence was monitored in growth medium supplemented with 20 μM methoxylated cypridina luciferin analog (MCLA (2-methyl-6-[*p*-methoxyphenyl]-3,7-dihydroimidazo [1,2-α]pyrazin-3-one); Invitrogen) using a luminometer (Lumicounter 2500) under the same conditions as for measuring [Ca^2+^]_cyt _[46].

To monitor H_2_O_2 _produced in extracellular medium, cells (80 mg mL^-1^) were washed and resuspended in 5 mM MES buffer (pH 7.0) containing 0.5 mM CaCl_2_, 0.5 mM K_2_SO_4 _and with or without 175 mM mannitol (Kurusu et al. 2005). A 25-μL aliquot of medium was mixed in a 96-well microtiter plate with 150 μl 50 mM Tris-HCl (pH 8.0) and 25 μL 0.462 mM luminol in 50 mM Tris-HCl, pH 8.0. Potassium ferricyanide (25 μL, 11.76 mM) was added, and H_2_O_2_-dependent chemiluminescence was recorded for 15 s using a luminometer (MicroLumat Plus LB96V, Berthold Technologies, Bad Wildbad, Germany).

### Statistical analysis

Statistical significance was determined using an unpaired Student's *t *test; *P *< 0.05 indicated significance.

## Abbreviations

BAPTA: 1,2-bis-(2-aminophenoxy)ethane-*N, N, N', N'*-tetra acetic acid; [Ca^2+^]_cyt_: cytosolic free Ca^2+ ^concentration; DIC: differential interference contrast; DPI: diphenylene iodonium; EGTA: ethylene glycol-bis-(2-aminoethylether)-*N, N, N', N'*-tetra acetic acid; GFP: green fluorescent protein; GUS: *β*-glucuronidase; H_2_O_2_: hydrogen peroxide; MAMP: microbe-associated molecular pattern; MCLA: 2-methyl-6-[*p*-methoxyphenyl]-3,7-dihydroimidazo [1,2-α]pyrazin-3-one; ^**•**^O_2_^-^: superoxide anion radical; MS medium: Murashige and Skoog medium; RACE: rapid amplification of cDNA ends; rlu: relative luminescence units; *RNAi*: RNA interference; RT: reverse transcriptase; ROS: reactive oxygen species; SHAM: salicylhydroxamic acid; TNP: trinitrophenol.

## Authors' contributions

TK, DN, and YY carried out most of the experiments and data analyses. TK and KK designed the study and wrote the manuscript. DN, YY and MG participated in confocal imaging analyses. MN, TY and KI carried out Ca^2+ ^uptake experiments in yeast. HH and HS participated in constructing transgenic lines and PCR analyses. YN, KS, HI participated in the design of the study and critically revised the manuscript. All authors read and approved the final manuscript.

## Authors' information

YN Present address: Laboratory of Cell Biology, Institute for Molecular and Cellular Regulation, Gunma University, Maebashi, Gunma 371-8510, Japan.

## Supplementary Material

Additional file 1 **Multiple amino acid sequence alignment of rice OsMCA1 and Arabidopsis MCA1 and MCA2 using Clustal W**. Asterisks indicate identical or conserved residues in the whole sequence in the alignment. Colons indicate conserved substitutions. Dots indicate semi-conserved substitutions. Light and dark gray box indicate the PLAC8 motif and coiled-coil motif, respectively. Two putative transmembrane segments (S1 and S2) are underlined. Click here for file

Additional file 2 **Expression of the *OsMCA1 *gene in rice tissues**. The expression of *OsMCA1 *in rice plants was determined by quantitative RT-PCR analysis. Total RNA was extracted from various tissues of rice plants as well as cultured cells. The amount of *OsMCA1 *mRNA was calculated from the threshold point in the log-linear range of the RT-PCR. The relative *OsMCA1 *mRNA level in cultured cells was standardized as 1. Data are means ± SD; n = 2-3 independent samples. Click here for file

Additional file 3 **Growth phenotype of *OsMCA1*-suppressed seedlings in MS medium**. Length of roots and shoots of the control line (20-5) and the *OsMCA1*-suppressed lines (20-2 and 38-5) of 10-days-old seedlings grown on MS medium plate in a growth chamber under long-day conditions (16 h light/8 h darkness, 28°C). Data are means ± SD; n = 7-10 independent seedlings. Click here for file

Additional file 4 **Effect of NADPH oxidase inhibitor on hypo-osmotic shock-induced ROS generation**. H_2_O_2 _concentration in extracellular medium was determined by ferricyanide-catalyzed oxidation of luminol. Diphenylene iodonium (DPI; 10 μM) was added to the rice cells 30 min before hypo-osmotic shock treatment. Data are the mean ± SE for five independent experiments for the wild type. ***P < 0.005; significantly different compared with the control. Click here for file

Additional file 5 **Effect of salicylhydroxamic acid, a peroxidase inhibitor, on hypo-osmotic shock-induced ROS generation**. The concentration of ^**•**^O_2_^- ^in extracellular medium was measured by MCLA chemiluminescence. Salicylhydroxamic acid (SHAM; 3 mM) was added to the rice cells 30 min before hypo-osmotic shock treatment. Average values and SE of three independent experiments for the wild type. Click here for file

Additional file 6 **Effect of *OsMCA1*-overexpression on hypo-osmotic shock-induced ROS generation**. H_2_O_2 _concentration in the extracellular medium was determined by ferricyanide-catalyzed oxidation of luminol. As a hypo-osmotic shock, growth medium was replaced by three-fold diluted medium at 0 min. Data are the mean ± SE for four independent experiments for two control lines (open circle for GUS No. 11; open triangle for GUS No. 7) and two overexpressor lines (closed diamond for OX No. 2; closed square for OX No. 3) are shown. *P < 0.05, significantly different compared with two control lines (GUS No. 7 and 11). Click here for file

## References

[B1] Reddy ASN Calcium: Silver bullet in signaling Plant Sci 2001 160 381 404 10.1016/S0168-9452(00)00386-1 11166425

[B2] Sanders D Pelloux J Brownlee C Harper JF Calcium at the crossroads of signaling Plant Cell 2002 14 S401 S417 12045291 10.1105/tpc.002899 PMC151269

[B3] Yang T Poovaiah BW Calmodulin-mediated signal network in plants Trends Plant Sci 2003 8 505 512 10.1016/j.tplants.2003.09.004 14557048

[B4] Fasano JM Massa GD Gilroy S Ionic signaling in plant responses to gravity and touch J Plant Growth Regul 2002 21 71 88 10.1007/s003440010049 12016507

[B5] Braam J Genome-wide identification of touch- and darkness-regulated Arabidopsis genes: a focus on calmodulin-like and *XTH *genes New Phytol 2005 165 373 389 15720654 10.1111/j.1469-8137.2004.01238.x

[B6] Toyoda M Furuichi T Tatsumi H Sokabe M Cytoplasmic calcium increases in response to change in the gravity vector in hypocotyls and petioles of Arabidopsis seedling Plant Physiol 2008 146 505 514 18055589 10.1104/pp.107.106450 PMC2245848

[B7] Dodd AN Kudla J Sanders D The language of calcium signaling Annu Rev Plant Biol 2010 2 593 620 10.1146/annurev-arplant-070109-104628 20192754

[B8] Véry AA Sentenac H Cation channels in the Arabidopsis plasma membrane Trends Plant Sci 2002 7 168 175 10.1016/S1360-1385(02)02262-8 11950613

[B9] White PJ Bowen HC Demidchik V Nichols C Davies JM Genes for calcium-permeable channels in the plasma membrane of plant root cells Biochim Biophys Acta 2002 1564 299 309 10.1016/S0005-2736(02)00509-6 12175911

[B10] Dutta R Robinson KR Identification and characterization of stretch-activated ion channels in pollen protoplasts Plant Physiol 2004 135 1398 1406 10.1104/pp.104.041483 15247410 PMC519057

[B11] Qi Z Kishigami A Nakagawa Y Iida H Sokabe M A mechanosensitive anion channel in *Arabidopsis thaliana *mesophyll cells Plant Cell Physiol 2004 45 1704 1708 10.1093/pcp/pch194 15574846

[B12] Telewski FW A unified hypothesis of mechanoperception in plants Am J Bot 2006 93 1466 1476 10.3732/ajb.93.10.1466 21642094

[B13] Haswell ES Peyronnet R Barbier-Brygoo H Meyerowitz EM Frachisse JM Two MscS homologs prvide mechanosensitive channel activities in the *Arabidopsis *root Curr Biol 2008 18 730 734 10.1016/j.cub.2008.04.039 18485707

[B14] Peyronnet R Haswell ES Barbier-Brygoo H Frachisse JM AtMSL9 and AtMSL10: Sensors of plasma membrane tension in Arabidopsis roots Plant Signal Behav 2008 3 726 729 10.4161/psb.3.9.6487 19704841 PMC2634572

[B15] Nakagawa Y Katagiri T Shinozaki K Qi Z Tatsumi H Furuichi T Kishigami A Sokabe M Kojima I Sato S Kato T Tabata S Iida K Terashima A Nakano M Ikeda M Yamanaka T Iida H *Arabidopsis *plasma membrane protein crucial for Ca^2+ ^influx and touch sensing in roots Proc Natl Acad Sci USA 2007 104 3639 3644 10.1073/pnas.0607703104 17360695 PMC1802001

[B16] Yamanaka T Nakagawa Y Mori K Nakano M Imamura T Kataoka H Terashima A Iida K Kojima I Katagiri T Shinozaki K Iida H MCA1 and MCA2 that mediate Ca^2+ ^uptake have distinct and overlapping roles in Arabidopsis Plant Physiol 2010 152 1284 1296 10.1104/pp.109.147371 20097794 PMC2832256

[B17] Zingarelli L Marré MT Massardi F Lado P Effects of hyperosmotic stress on K^+ ^fluxes, H^+ ^extrusion, transmembrane electric potential difference and comparison with the effects of fusicoccin Physiol Plant 1999 106 287 295 10.1034/j.1399-3054.1999.106305.x

[B18] Beffagna N Buffoli B Busi C Modulation of reactive oxygen species production during osmotic stress in *Arabidopsis thaliana *cultured cells: involvement of the plasma membrane Ca^2+^-ATPase and H^+^-ATPase Plant Cell Physiol 2005 46 1326 1339 10.1093/pcp/pci142 15937326

[B19] Rouet MA Mathieu Y Barbier-Brygoo H Laurière C Characterization of active oxygen-producing proteins in response to hypo-osmolarity in tobacco and Arabidopsis cell suspensions: identification of a cell wall peroxidase J Exp Bot 2006 57 1323 1332 10.1093/jxb/erj107 16551688

[B20] Hayashi T Harada A Sakai T Takagi S Ca^2+ ^transient induced by extracellular changes in osmotic pressure in Arabidopsis leaves: differential involvement of cell wall-plasma membrane adhesion Plant Cell Environ 2006 29 661 672 10.1111/j.1365-3040.2005.01447.x 17080616

[B21] Moran N Osmoregulation of leaf motor cells FEBS Lett 2007 581 2337 2347 10.1016/j.febslet.2007.04.002 17434488

[B22] Cazalé AC Rouet-Mayer MA Barbier-Brygoo H Mathieu Y Laurière C Oxidative burst and hypoosmotic stress in tobacco cell suspensions Plant Physiol 1998 116 659 669 10.1104/pp.116.2.659 9490766 PMC35124

[B23] Kaku H Nishizawa Y Ishii-Minami N Akimoto-Tomiyama C Dohmae N Takio K Minami E Shibuya N Plant cells recognize chitin fragments for defense signaling through a plasma membrane receptor Proc Natl Acad Sci USA 2006 103 11086 11091 10.1073/pnas.0508882103 16829581 PMC1636686

[B24] Miya A Albert P Shinya T Desaki Y Ichimura K Shirasu K Narusaka Y Kawakami N Kaku H Shibuya N CERK1, a LysM receptor kinase, is essential for chitin elicitor signaling in *Arabidopsis* Proc Natl Acad Sci USA 2007 104 19613 19618 10.1073/pnas.0705147104 18042724 PMC2148337

[B25] Trewavas A Knight M Mechanical signalling, calcium and plant form Plant Mol Biol 1994 26 1329 1341 10.1007/BF00016478 7858194

[B26] Ji XM Raveendran M Oane R Ismail A Lafitte R Bruskiewich R Cheng SH Bennett J Tissue-specific expression and drought responsiveness of cell-wall invertase genes of rice at flowering Plant Mol Biol 2005 59 945 964 10.1007/s11103-005-2415-8 16307368

[B27] Brady KU Kruckeberg AR Bradshaw HD Jr Evolutionary ecology of plant adaptation to serpentine soils Annu Rev Ecol Evol Syst 2005 36 243 266 10.1146/annurev.ecolsys.35.021103.105730

[B28] Ogasawara Y Kaya H Hiraoka G Yumoto F Kimura S Kadota Y Hishinuma H Senzaki E Yamagoe S Nagata K Nara M Suzuki K Tanokura M Kuchitsu K Synergistic activation of the Arabidopsis NADPH oxidase AtrbohD by Ca^2+ ^and phosphorylation J Biol Chem 2008 283 8885 8892 10.1074/jbc.M708106200 18218618

[B29] Takeda S Gapper C Kaya H Bell E Kuchitsu K Dolan L Local positive feedback regulation determines cell shape in root hair cells Science 2008 319 1241 1244 10.1126/science.1152505 18309082

[B30] Kimura S Kaya H Kawarazaki T Hiraoka G Senzaki E Michikawa M Kuchitsu K Protein phosphorylation is a prerequisite for the Ca^2+^-dependent activation of *Arabidopsis *NADPH oxidases and may function as a trigger for the positive feedback regulation of Ca^2+ ^and reactive oxygen species Biochim Biophys Acta 2011 1823 398 405 10.1016/j.bbamcr.2011.09.011 22001402

[B31] Monshausen GB Bibikova TN Weisenseel MH Gilroy S Ca^2+ ^regulates reactive oxygen species production and pH during mechanosensing in Arabidopsis roots Plant Cell 2009 21 2341 2356 10.1105/tpc.109.068395 19654264 PMC2751959

[B32] Kurusu T Yagala T Miyao A Hirochika H Kuchitsu K Identification of a putative voltage-gated Ca^2+ ^channel as a key regulator of elicitor-induced hypersensitive cell death and mitogen-activated protein kinase activation in rice Plant J 2005 42 798 809 10.1111/j.1365-313X.2005.02415.x 15941394

[B33] Murashige T Skoog F A revised medium for rapid growth and bioassays with tobacco tissue cultures Physiol Plant 1962 15 473 497 10.1111/j.1399-3054.1962.tb08052.x

[B34] Kuchitsu K Kikuyama M Shibuya N *N*-acetylchitooligosaccharides, biotic elicitors for phytoalexin production, induce transient membrane depolarization in suspension-cultured rice cells Protoplasma 1993 174 79 81 10.1007/BF01404046

[B35] Kojima S Takahashi Y Kobayashi Y Monna L Sasaki T Araki T Yano M *Hd3a*, a rice ortholog of the Arabidopsis *FT *gene, promotes transcription to flowering downstream of *Hd1 *under short-day conditions Plant Cell Physiol 2002 43 1096 1105 10.1093/pcp/pcf156 12407188

[B36] Kurusu T Hamada J Nokajima H Kitagawa Y Kiyoduka M Takahashi A Hanamata S Ohno R Hayashi T Okada K Koga J Hirochika H Yamane H Kuchitsu K Regulation of microbe-associated molecular pattern-induced hypersensitive cell death, phytoalexin production and defense gene expression by calcineurin B-like protein-interacting protein kinases, OsCIPK14/15, in rice cultured cells Plant Physiol 2010 153 678 692 10.1104/pp.109.151852 20357140 PMC2879771

[B37] Karimi M Inzé D Depicker A GATEWAY vectors for Agrobacterium-mediated plant transformation Trends Plant Sci 2002 7 193 195 10.1016/S1360-1385(02)02251-3 11992820

[B38] Miki D Shimamoto K Simple RNAi vectors for stable and transient suppression of gene function in rice Plant Cell Physiol 2004 45 490 495 10.1093/pcp/pch048 15111724

[B39] Tanaka H Kayano T Ugaki M Shiobara F Onodera H Ono K Tagiri A Nishizawa Y Shibuya N Ultra-fast transformation technique for monocotyledons. International Patent Application 2001 No. WO 01/06844 A1

[B40] Fuse T Sasaki T Yano M Ti-plasmid vectors useful for functional analysis of rice genes Plant Biotechnol 2001 18 219 222 10.5511/plantbiotechnology.18.219

[B41] Knight MR Campbell AK Smith SM Trewavas AJ Transgenic plant aequorin reports the effects of touch and cold-shock and elicitors on cytoplasmic calcium Nature 1991 352 524 526 10.1038/352524a0 1865907

[B42] Igasaki T Ishida Y Mohri T Ichikawa H Shinohara K Transformation of *Populus alba *and direct selection of transformants with the herbicide bialaphos Bulletin of FFPRI 2002 1 235 240

[B43] An G High efficiency transformation of cultured tobacco cells Plant Physiol 1985 79 568 570 10.1104/pp.79.2.568 16664453 PMC1074928

[B44] Kurusu T Hamada H Sugiyama Y Yagala T Kadota Y Furuichi T Hayashi T Umemura K Komatsu S MIyao A Hirochika H Kuchitsu K Negative feedback regulation of microbe-associated molecular pattern-induced cytosolic Ca^2+ ^transients by protein phosphorylation J Plant Res 2011 124 415 424 10.1007/s10265-010-0388-4 21063744

[B45] Iida H Yagawa Y Anraku Y Essential role for induced Ca^2+ ^influx followed by [Ca^2+^]_i _rise in maintaining viability of yeast cells late in the mating pheromone response pathway. A study of [Ca^2+^]_i _in single *Saccharomyces cerevisiae *cells with imaging of fura-2 J Biol Chem 1990 265 13391 13399 2198292

[B46] Kurusu T Hamada H Hanamata S Kuchitsu K Roles of calcineurin B-like protein-interacting protein kinases in plant innate immunity in rice Plant Signal Behav 2010 5 1045 1047 10.4161/psb.5.8.12407 20724838 PMC3115194

