# Supplementary figures and images for: Plasma membrane protein OsMCA1 is involved in regulation of hypo-osmotic shock-induced Ca2+ influx and modulates generation of reactive oxygen species in cultured rice cells
Source: BMC Plant Biol. 2012 Jan 23;12:11. doi: 10.1186/1471-2229-12-11 (PMC3313898; doi:10.1186/1471-2229-12-11)

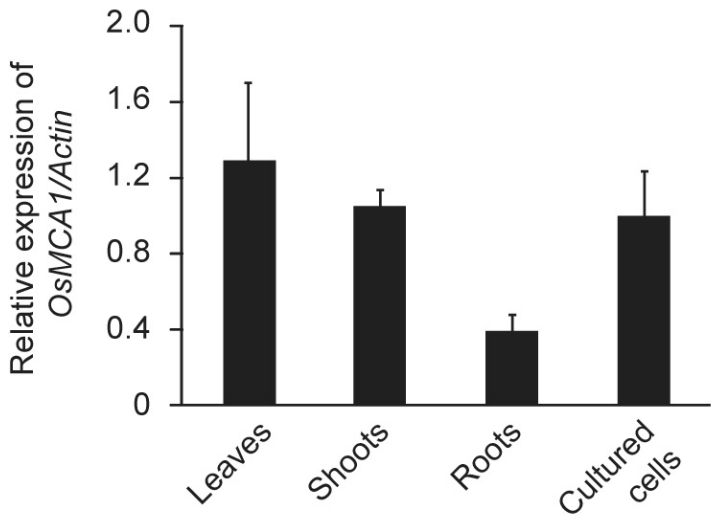

Supplement: Additional file 2 — Expression of the OsMCA1 gene in rice tissues. The expression of OsMCA1 in rice plants was determined by quantitative RT-PCR analysis. Total RNA was extracted from various tissues of rice plants as well as cultured cells. The amount of OsMCA1 mRNA was calculated from the threshold point in the log-linear range of the RT-PCR. The relative OsMCA1 mRNA level in cultured cells was standardized as 1. Data are means ± SD; n = 2-3 independent samples. [file 1471-2229-12-11-S2.PDF]

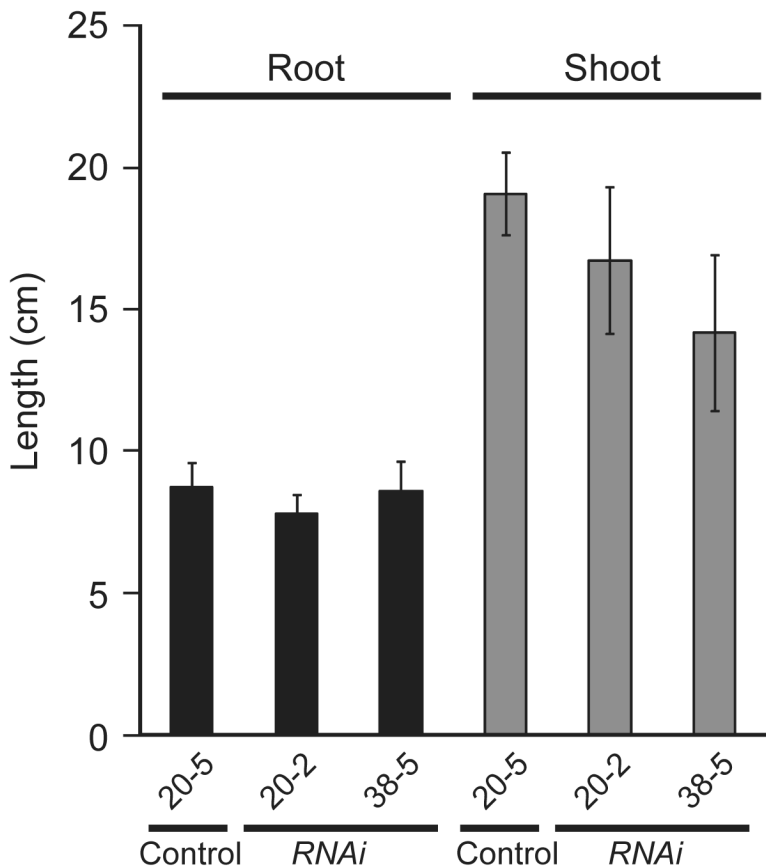

Supplement: Additional file 3 — Growth phenotype of OsMCA1-suppressed seedlings in MS medium. Length of roots and shoots of the control line (20-5) and the OsMCA1-suppressed lines (20-2 and 38-5) of 10-days-old seedlings grown on MS medium plate in a growth chamber under long-day conditions (16 h light/8 h darkness, 28°C). Data are means ± SD; n = 7-10 independent seedlings. [file 1471-2229-12-11-S3.PDF]

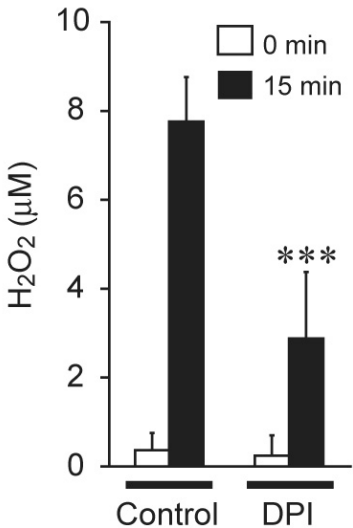

Supplement: Additional file 4 — Effect of NADPH oxidase inhibitor on hypo-osmotic shock-induced ROS generation. H2O2 concentration in extracellular medium was determined by ferricyanide-catalyzed oxidation of luminol. Diphenylene iodonium (DPI; 10 μM) was added to the rice cells 30 min before hypo-osmotic shock treatment. Data are the mean ± SE for five independent experiments for the wild type. ***P < 0.005; significantly different compared with the control. [file 1471-2229-12-11-S4.PDF]

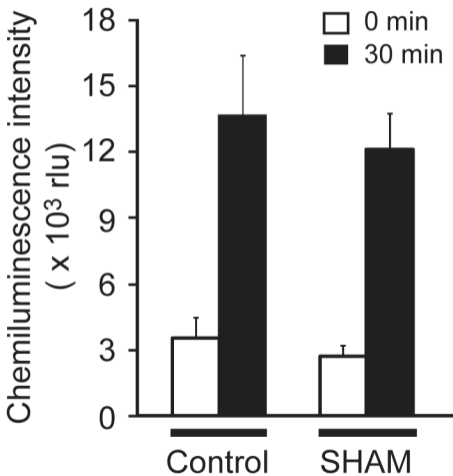

Supplement: Additional file 5 — Effect of salicylhydroxamic acid, a peroxidase inhibitor, on hypo-osmotic shock-induced ROS generation. The concentration of •O2- in extracellular medium was measured by MCLA chemiluminescence. Salicylhydroxamic acid (SHAM; 3 mM) was added to the rice cells 30 min before hypo-osmotic shock treatment. Average values and SE of three independent experiments for the wild type. [file 1471-2229-12-11-S5.PDF]

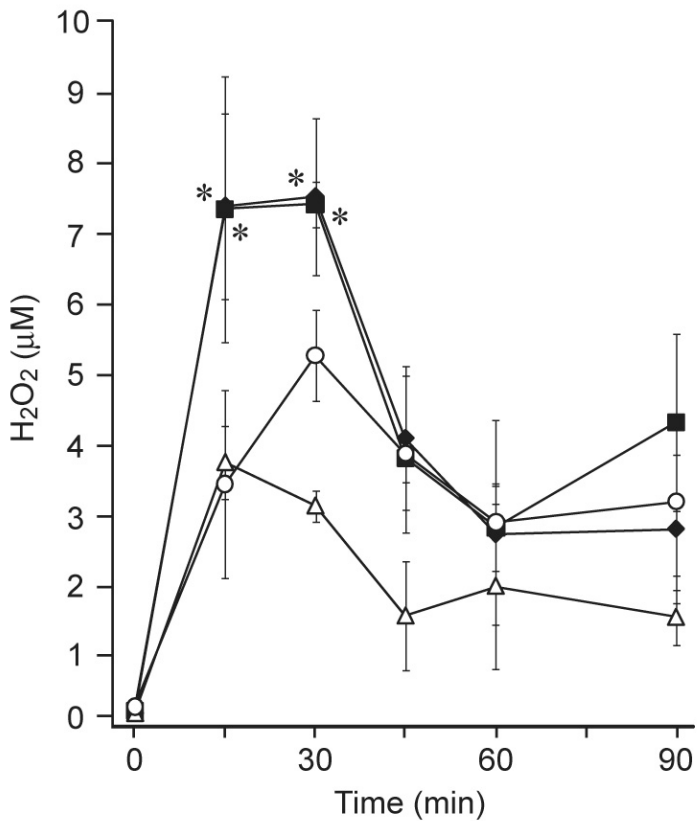

Supplement: Additional file 6 — Effect of OsMCA1-overexpression on hypo-osmotic shock-induced ROS generation. H2O2 concentration in the extracellular medium was determined by ferricyanide-catalyzed oxidation of luminol. As a hypo-osmotic shock, growth medium was replaced by three-fold diluted medium at 0 min. Data are the mean ± SE for four independent experiments for two control lines (open circle for GUS No. 11; open triangle for GUS No. 7) and two overexpressor lines (closed diamond for OX No. 2; closed square for OX No. 3) are shown. *P < 0.05, significantly different compared with two control lines (GUS No. 7 and 11). [file 1471-2229-12-11-S6.PDF]
