# Supplementary material for: Plasma membrane protein OsMCA1 is involved in regulation of hypo-osmotic shock-induced Ca2+ influx and modulates generation of reactive oxygen species in cultured rice cells
Source: BMC Plant Biol. 2012 Jan 23;12:11. doi: 10.1186/1471-2229-12-11 (PMC3313898; doi:10.1186/1471-2229-12-11)
Supplement: Additional file 1 — Multiple amino acid sequence alignment of rice OsMCA1 and Arabidopsis MCA1 and MCA2 using Clustal W. Asterisks indicate identical or conserved residues in the whole sequence in the alignment. Colons indicate conserved substitutions. Dots indicate semi-conserved substitutions. Light and dark gray box indicate the PLAC8 motif and coiled-coil motif, respectively. Two putative transmembrane segments (S1 and S2) are underlined. [file 1471-2229-12-11-S1.PDF]

```
OsMCA1   MA-SWENLGDVATVVQLTGLDAVRLISMIVKAASTARLHKRNCRRFAQHLKLIGGLLEQL
MCA1     MSHSWDGLGEIASVAQLTGLDAVKLIGLIVKAANTAWMHKKNCRQFAQHLKLIGNLLEQL
MCA2     MANSWDQLGEIASVAQLTGIDALKLIGMIVNAANTARMHKKNCRQFAHHLKLIRNLLEQI
         *: **: **::*:*.****:**::**.:**:**.** :**:***:**:***** .****:

OsMCA1   RVSELKKYPETREPLEQLEDALRRAYLLVHSCQDRSYLYLLAMGWNIVYQFRKAQNEIDN
MCA1     KISEMKKYPETREPLEGLEDALRRSYLLVNSCRDRSYLYLLAMGWNIVYQFRKHQDEIDR
MCA2     KNSEMNQRSEILEPLQGLDDALRRSYILVKSCQEKSYLYLLAMGWNIVNQFEKAQNEIDL
         : **::: .*  ***: *:*****:*:**:**:::************* **.* *:***

OsMCA1   YLRLVPLITLVDNARVRERMEYIERDQCEYSFDDEDKEVQDALLNPDPS-TNPTVVLKKT
MCA1     FLKIIPLITLVDNARIRERFEYIDRDQREYTLDEEDRHVQDVILKQESTREAAS-VLKKT
MCA2     FLKIVPLINMADNARIRERLEAIERDQREYTLDEEDRKVQDVILKQESTREAATSVLKKT
         :*:::***.:.****:***:* *:*** **::*:**:.***.:*: :.:   .: *****

OsMCA1   LSCSYPNLPFNEALRKESEKLQVELQRSQSNMDMGQCEVIQHLLGVTKTVASSIP--EKC
MCA1     LSCSYPNLRFCEALKTENEKLQIELQRSQEHYDVAQCEVIQRLIGVTQAAAAVEPDSEKE
MCA2     LSRSYPNMGFCEALKTEEEKLQLELQRSRARYDADQCEVIQRLIDVTQTAATVEPNLEKV
         ** ****: * ***:.*.****:*****: . *  ******:*:.**::.*:  *  **

OsMCA1   ATPKVSEKADSNHTKVSEDSAKTYHDDSPKKQKDACTAPRSSPPSSYGHDLVSSRG---S
MCA1     LTKKASKKSERSSSMKTEY---SYDEDSPKK-SSTRAASRSTSNVSSGHDLLSRRASQAQ
MCA2     LTKKE----ELTSSKKRDD---LYDTDS----SSIRADSRSTSYVSSGHELLSGRS--LQ
          * *     : . :   :     *. **    ..  : .**:.  * **:*:* *.   .

OsMCA1   YSDEWHADLLGCCSEPSLCLRTFFFPCGTFSKIASIAKNRPMSSSEACNDIMAYSLILSC
MCA1     HHEEWHTDLLACCSEPSLCFKTFFFPCGTLAKIATAASNRHISSAEACNELMAYSLILSC
MCA2     HRGNWHADLLDCCSEPCLCLKTLFFPCGTLAKISTVATSRQISSTEVCKNLIVYSLILSC
         :  :**:*** *****.**::*:******::**:: *..* :**:*.*::::.*******
                                             S1

OsMCA1   CCYTCCVRRKLRQKLNIAGGCIDDFLSHLMCCCCALVQEWREVEIR-GAYG-ERT--KIS
MCA1     CCYTCCVRRKLRKTLNITGGFIDDFLSHVMCCCCALVQELREVEIR-GAYGTEKT--KIS
MCA2     CCYTCCIRKKLRKTLNITGGCIDDFLSHLMCCCCALVQELREVEIHRASYGTEKSNKEMS
         ******:*:***:.***:** *******:********** *****: .:** *::  ::*
               S2

OsMCA1   PPSFQYMEH
MCA1     PPSSQFMEH
MCA2     PPTPQFMEE
         **: *:**.
```
